# Evaluation of a Breath-Indicating Device for Enhanced Respiratory Monitoring and Apnea Detection in Postoperative Care: A Comparative Study

**DOI:** 10.7759/cureus.81846

**Published:** 2025-04-07

**Authors:** Dylan Rowe, Mariam Rowe, Dylan Stubbs, Chase Pontifex, Phillip Melksham

**Affiliations:** 1 School of Medicine, Griffith University, Brisbane, AUS; 2 Faculty of Medicine, The University of Queensland, Brisbane, AUS; 3 School of Medicine, Griffith University, Gold Coast, AUS; 4 Department of Anesthesia, Wesley Medical Center, Brisbane, AUS

**Keywords:** apnea detection, breath-indicating device, continuous monitoring, perioperative care, respiratory monitoring

## Abstract

Objectives: Accurate respiratory monitoring is crucial in post-anesthetic care settings due to increased risks of respiratory complications. This study evaluates the impact of a new breath-indicating device, ApnoLight (PEMDx Pty Ltd, Brisbane, Queensland, Australia), on medical staff's accuracy in recording respiratory rates and detecting apneic events, along with the device's acceptance among nurses.

Methods: Twenty-five nurses from a hospital in Brisbane, Australia, participated. A simulated patient was fitted with the ApnoLight device on an oxygen mask. Nurses conducted six respiratory rate observations at varying distances (bedside, two meters, and five meters), both with and without the device. The patient's respiratory rates varied from eight to 25 breaths per minute. The accuracy of respiratory rate recordings and the time to identify apnea events were compared between simple observation and device-assisted observations.

Results: The ApnoLight significantly reduced error rates in respiratory rate recordings: 27.58% at the bedside (P = 0.09, t = 1.31), 90.99% at two meters (P = 0.02, t = 1.98), and 96.37% at five meters (P = 0.0002, t = 4.02). The mean time to identify apnea decreased from 12.96 ± 9.12 seconds (simple observation) to 7.42 ± 2.19 seconds (with ApnoLight device). All apnea events were identified with the device, whereas four were undetected without it. Feedback showed that 96% (N = 24) of nurses found the device improved respiratory rate accuracy, and 100% (N = 25) found it made apnea identification easier.

Conclusions: The ApnoLight device has the potential to enhance respiratory rate monitoring accuracy and apnea detection in postoperative settings. Its implementation could improve patient safety and streamline clinical workflows.

## Introduction

Accurate ventilation monitoring is a fundamental yet challenging component of patient care, especially in post-anesthesia care settings. Patients may be at risk of depressed respiration due to a variety of factors, such as the surgical procedure itself, post-surgical discomfort, sedation from medications, ineffective airway management, or impaired gas exchange [1,2]. This can lead to severe complications, including atelectasis, pneumonia, acute respiratory failure, pulmonary edema, and exacerbation of chronic pulmonary conditions [3-5]. Additionally, deviations in respiratory rates can be an early indicator of major clinical events such as cardiac arrest, sepsis, and respiratory infections, with delayed detection contributing significantly to perioperative complications and mortality [6,7].

Traditionally, respiratory rate measurement involves auscultation or simple visual observation over 60 seconds, as recommended by the World Health Organization (WHO) [8]. However, this method in busy clinical environments often leads to inaccuracies and inconsistencies, where counting chest wall movements is prone to error [9-11]. The need for a more reliable monitoring system is evidently a clinical need. European anesthesiology professional societies have highlighted the importance of continuous monitoring of oxygenation and ventilation in their Helsinki Declaration on Patient Safety in Anesthesiology, advocating for capnography if hypoventilation is anticipated [12,13]. Despite existing recommendations, capnography is often underutilized in clinical practice due to practical limitations, leading to a growing body of research focused on the development of portable monitoring devices [14].

Recent technological advancements offer promising alternatives for respiratory rate monitoring. Innovations such as monitoring of thoracic micromovements based on the principles of seismocardiography, ballistocardiography, remote camera recordings, and deployment of integrated optical fibers have been extensively studied in controlled settings [15]. Although these technologies have demonstrated potential benefits, their adoption into clinical practice remains limited. This is partly because many of these technologies were designed for different applications, such as monitoring mechanically ventilated patients or those with obstructive sleep apnea, rather than for spontaneous ventilation in awake patients [16-19]. Furthermore, an absence of large-scale, randomized controlled trials validating these newer technologies in diverse clinical environments has been identified. Information regarding the practitioner's perception of the impact respiratory monitoring devices have on existing workflow appears to be underrepresented in the literature.

While advancements in respiratory monitoring technologies show promise, there remains a critical need for solutions that can be seamlessly integrated into current clinical practice. Addressing the limitations of current methods and further validating emerging technologies through comprehensive trials will be essential for enhancing patient safety and improving outcomes in postoperative care environments [20].

A new prototype device called the ApnoLight (PEMDx Pty Ltd, Brisbane, Queensland, Australia) has been recently patented and designed to non-invasively, inexpensively, and portably indicate patient respiration and apnea. It consists of a small electronic circuit board containing a combined temperature and humidity integrated sensor and a microcontroller running a breath detection algorithm. The electronics are protected in a semi-translucent plastic enclosure, with a piece of adhesive foam on its backing. The device adheres to the exterior of any standard oxygen face mask, overlapping the side ventilation holes. It senses exhaled gases as they exit the oxygen mask to accurately determine when a breath has occurred; it then indicates one green light flash per breath or a continuously flashing red light during apnea.

We designed this study to examine the impact of the device on medical staff's accuracy when manually recording a patient's respiratory rate or attempting to determine if the patient was having an apneic event. We also aim to determine the acceptance of this device in the current workflow of the postoperative care setting among nurses. To our knowledge, this is the first clinical test of this new technology exclusively performed by medical staff in a clinical setting to specifically address these aims.

## Materials and methods

Participants

Twenty-five nurses were recruited from one hospital in Brisbane, Australia. This study was approved by the institutional Uniting Care Human Research Ethics Committee, and written informed consent was obtained from all participants. The study adhered to the Enhancing the Quality and Transparency of Health Research (EQUATOR) guidelines and was conducted in accordance with the principles of the Declaration of Helsinki under the supervision of the principal investigator. A member of the medical staff was recruited from the same location to act as a simulated patient; this person was a 57-year-old man with a body mass index (BMI) of 28 kg/m^2^ and no known comorbidities.

Mask setup

**Figure 1:**
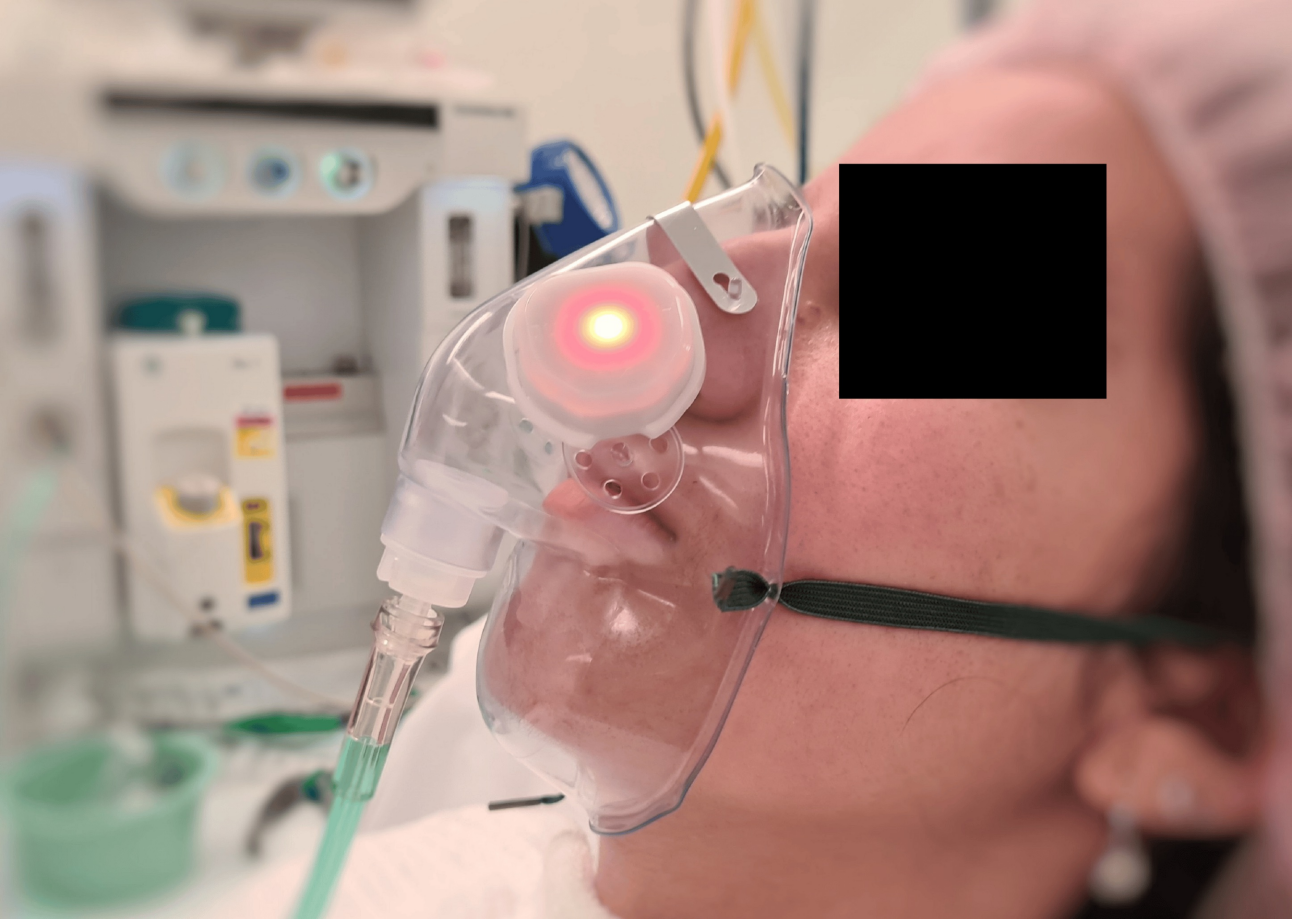
ApnoLight shown attached to the exterior of the oxygen face mask overlapping the ventilation holes, flashing red to indicate an apneic event

The ApnoLight consists of a single electronic unit contained within a plastic enclosure, with an adhesive foam on its backing. Following the manufacturer's instructions for use, the device was adhered to the exterior of an elongated, medium-concentration mask (Parker Healthcare, Melbourne, Australia), overlapping the ventilation holes (Figure 1).

A carbon dioxide (CO2) gas sampling line (Parker Healthcare, Melbourne, Australia) was then connected from a GE Healthcare capnography monitor (GE Medical Systems Information Technologies INC, WI, USA) to the opposite side of the face mask. The simulated patient was then fitted with the face mask with the attached ApnoLight and capnography gas sample lines, and oxygen was set to 6 L per minute. The ApnoLight provides one green light indication for each breath and a continuously flashing red light indication during apnea. The device utilizes a combination of temperature and humidity measurements of the exhaled air as it exits the oxygen mask ventilation holes.

Experimental protocol

The study was conducted in a postoperative recovery room designed to replicate real clinical environments and conditions. Each nurse received training on the use of ApnoLight, followed by an explanation of the study protocol. Each nurse completed a total of six respiratory rate observations, using one minute for each measurement. The observations were conducted at the bedside, two meters from the bedside, and five meters from the bedside, first with simple observation without any devices and then repeated with the addition of the ApnoLight. The simulated patient was permitted to breathe through their oral and/or nasal passageway with a tidal volume they considered appropriate. The patient controlled their respiratory rate using a metronome via wireless headphones, with rates ranging from eight to 25 breaths per minute. The CO2 monitor was used to confirm that the simulated patient was accurately breathing at the rate set by the metronome. The specific rates were randomly varied for each test, which were controlled and recorded by the study facilitator. During each test, the simulated patient held their breath to indicate an apnea event when the metronome was paused by the facilitator. The time taken for the nurse to recognize and alert the facilitator of the apnea was recorded both with and without the device. Apnea events were completed only during the two-meter assessments to ensure consistency; however, nurses were blinded to this and informed that apnea may occur at any time or distance. Nurses recorded the respiratory rate at each distance under both conditions (simple observation and device-assisted) on a paper form. Nurses also completed a survey at the end of the study to provide feedback on the usability and effectiveness of the device. Testing and data collection were performed by a doctor who was trained in the study protocol.

Data analysis

Participant characteristics were tabulated and analyzed to determine the mean, standard deviation, and P-values of the study group. Data were analyzed using standard statistical methods to compare the accuracy of respiratory rate monitoring with and without the ApnoLight. Paired t-tests were employed to assess differences in respiratory rate error between the two methods, where statistical significance was set at P < 0.05.

## Results

We enrolled 25 nurses in this study aged between 20 and 59 with experience levels ranging from one to 13+ years and a simulated patient that remained consistent throughout. Descriptive statistics for the study participants are presented in Table 1. All 25 participants completed the study, and all data was included in our analysis. There were no equipment malfunctions or errors during the study.

**Table 1:** Descriptive statistics for the study participants

Age range	Number of nurses (N)
20-29	4
30-39	9
40-49	8
50-59	4
Years of experience	Number of nurses (N)
0-3	1
4-6	5
7-9	2
10-12	4
13+	13

Primary end point: performance

We found that the rate of error in respiratory rate recordings using observation and examination improved when using ApnoLight by 27.58% (P = 0.09, t = 1.31) at the bedside, 90.99% (P = 0.02, t = 1.98) at two meters, and 96.27% (P = 0.0002, t = 4.02) at five meters from the patient. The mean error (breaths per minute) and standard deviation with and without the device when compared to the capnography recordings are shown in Table 2.

**Table 2:** Mean error (SD) with and without ApnoLight when compared to the capnography recordings Data are presented as mean ± SD. P-values are calculated using paired t-tests. SD: standard deviation

Distance from the patient	Error without ApnoLight (mean ± SD)	Error with ApnoLight (mean ± SD)	Error reduction (%)	t-value	P-value
Bedside	1.16 ± 1.74	0.84 ± 1.12	27.58%	1.31	0.09
2 meters	11.11 ± 3.40	1.00 ± 1.30	90.99%	1.98	0.02
5 meters	23.19 ± 4.91	0.84 ± 1.33	96.37%	4.02	0.0002

Our study found that the mean time to identify apnea was 12.96 ± 9.12 seconds when using simple observation without ApnoLight, which improved to 7.42 ± 2.19 seconds when using the device. Additionally, four apnea events were not identified by the participants when the device was not used, but all apnea events were identified by the participants when the device was used. This data is shown in Table 3.

**Table 3:** Mean (SD) time for participants to identify apnea and the number of apnea events identified Data are presented as mean ± SD. SD: standard deviation

	Time to identify apnea (mean ± SD) (seconds)	Number of apnea events identified	Number of apnea events missed
Without ApnoLight	12.96 ± 9.12	84% (N = 21)	16% (N = 4)
With ApnoLight	7.42 ± 2.19	100% (N = 25)	0% (N = 0)

Secondary end point: survey feedback

**Figure 2:**
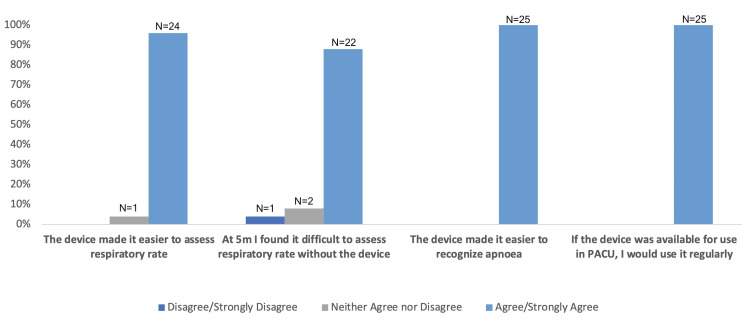
Participants' post-test survey results N: number of respondents

The participants rated the device highly useful, with 96% (N = 24) agreeing that it improved their ability to accurately measure a patient's respiratory rate. All participants agreed that the device made apnea easier to identify, and they would use it if available in the postoperative care environment. These secondary end points are shown in Figure 2.

## Discussion

Our study aimed to evaluate the effectiveness and accepted use of an electronic breath-indicating device in enhancing the accuracy of manual respiratory rate monitoring and the timely recognition of apnea events by nurses in a postoperative recovery room setting. The results demonstrated a significant improvement in both respiratory rate accuracy and apnea recognition when the study participants used the ApnoLight device compared to simple observation alone.

Previous research has shown that a combination of respiratory monitors such as capnography and pulse oximetry monitoring in a postoperative care unit is more beneficial than pulse oximetry alone. However, it is still not routinely utilized due to a range of reasons, including the time required for setup, bulky monitoring equipment, or lack of funding for a 1:1 ratio of capnography machines to patients [21]. Enhanced postoperative respiratory monitoring can be achieved with a user-friendly, affordable, and reliable device. This study provides the initial step toward implementing a new technology to enhance respiratory monitoring ability in the post-anesthesia care unit and ambulatory setting or on patient wards.

In this study, ApnoLight reduced the error in respiratory rate measurements across all distances examined. At the bedside, the error rate was reduced by 27.58%, which was not statistically significant (P = 0.09, t = 1.31). However, at two meters and five meters, the error rates were significantly reduced by 90.99% (P = 0.02, t = 1.98) and 96.37% (P = 0.0002, t = 4.02), respectively. These findings suggest that the device is particularly beneficial in scenarios where the healthcare professional tasked with monitoring a patient's respiratory rate is not near the patient, thereby enhancing the monitoring capability in a busy or spatially constrained environment.

The mean time to identify apnea was reduced from 12.96 seconds with simple observation to 7.42 seconds with the breath-indicating device. Additionally, four apnea events were missed without the device, but all apnea events were identified when the device was used. These results highlight the device's potential to improve patient safety by enabling quicker and more reliable detection of critical events such as apnea, which is essential in a postoperative setting where prompt intervention has been shown to improve patient outcomes [22].

The qualitative feedback from the participants suggested that a visual indicator of respiration would aid in the clinical management of a patient. From the post-use survey, 96% (N = 24) of the nurses agreed that the device improved their ability to accurately measure respiratory rates, and 100% (N = 25) indicated that it made apnea easier to identify. This high level of acceptance suggests that there is potential to introduce this technology into the current postoperative setting without workflow disruption.

Several limitations have been identified in the study. Firstly, it was conducted in a simulated environment with a single simulated patient, which may not replicate the variability encountered in clinical practice when other distractions and confounding factors are present. Studies have also shown that health practitioners do not always utilize a full 60 seconds when completing a respiratory rate assessment or strictly follow the WHO guidelines [23]. Being forced to assess for the full 60 seconds in this study may have artificially increased the assessment accuracy when not using ApnoLight. Additionally, the small sample size of 25 nurses, while sufficient for demonstrating statistical significance in this study, may limit the generalizability of the findings. A final limitation is that participants were aware of the purpose of the study, and the protocol was neither randomized nor double-blinded, with the exception of blinding of the timing of the apnea event, which introduces confounding factors and reduces the dependability of the study results.

Future studies should aim to include a larger and more diverse sample of nurses and patients across multiple healthcare settings.

Further research is needed to explore the long-term impacts of integrating a breath-indicating device into routine clinical practice, including its effects on patient outcomes and nursing workflow. Additionally, studies should investigate the cost-effectiveness of the device and its potential to reduce the incidence of postoperative complications and improve patient outcomes in recovery. Exploring the integration of the device into other hospital settings could also provide valuable insights into optimizing patient care on a broader basis.

## Conclusions

In conclusion, we evaluated the accuracy of simple respiratory observations both with and without the ApnoLight breath-indicating device. Our study demonstrates a notable reduction in respiratory rate errors and an improved ability of study participants to promptly identify apnea when using the ApnoLight device. This non-intrusive technology can improve vital sign monitoring by accurately detecting respiratory rate and apneic events. It has the potential to eliminate the need for costly and complex anesthetic equipment and help prevent respiratory emergencies, leading to shorter hospital stays, and reduce the overall cost of patient care. Furthermore, this device has potential applications in low-resource or emergency situations, such as natural disasters, epidemics, humanitarian crises, or military conflicts, where the need is for portable, lightweight, durable, user-friendly, easily replaceable, and cost-effective equipment. To fully establish the significance of our findings, additional comparative studies involving a larger and more diverse patient population, including pediatric, adult, and geriatric patients with cardiac and pulmonary comorbidities, are necessary to further evaluate the device's ability to accurately monitor respiration and apnea.
